# Special Issue: Prostate Cancer Research Update: Molecular Diagnostic Biomarkers

**DOI:** 10.3390/ijms27209311

**Published:** 2026-10-20

**Authors:** Mikhail Baryshev

**Affiliations:** Institute of Microbiology and Virology, Riga Stradins University, Ratsupites Str 5, LV-1067 Riga, Latvia; mihails.barisevs@rsu.lv

## 1. Integrating Molecular Diagnostics, Epigenetics, Genomics, and Theranostics in Prostate Cancer

Prostate cancer remains a major health burden and presents a particular challenge for precision oncology because of its substantial molecular heterogeneity and variable clinical behavior. Its incidence is expected to increase by more than 35% in the coming decades [1,2]. The global burden from the disease is anticipated to increase to 1.7 million new cases and 499,000 deaths by 2030 [3]. More recent IARC projections estimate about 1.83 million prostate cancer cases in 2030, rising to approximately 2.88 million by 2050. Patients may present with localized disease that remains indolent for many years, whereas others develop metastatic disease and, ultimately, metastatic castration-resistant prostate cancer (mCRPC), in which treatment resistance and clonal evolution become major determinants of outcome [4]. Prostate cancer is increasingly characterized through integrated molecular, epigenetic, genomic, imaging, and computational approaches that complement conventional prostate-specific antigen (PSA)-based assessment.

This Special Issue of the International Journal of Molecular Sciences brings together six complementary contributions spanning precision diagnostics, allele-specific DNA methylation, heterogeneous methylation analysis in liquid biopsy, androgen receptor (AR)-related genomic diversity, longitudinal biomarker-guided management of metastatic disease, and prostate-specific membrane antigen (PSMA)-targeted ligand chemistry. As shown in Graphical overview, the six contributions in this Special Issue illustrate the transition from conventional assessment toward integrated molecular medicine. They address distinct scientific questions, but collectively trace a continuum from detection and biological characterization to longitudinal monitoring, treatment adaptation, and theranostic development.

The review by Vakili et al. provides the broad diagnostic framework for the Special Issue by examining contemporary innovations in prostate cancer diagnosis, treatment, and future directions [5]. It places established and emerging biomarkers within a broader diagnostic ecosystem that includes imaging, liquid biopsy, molecular assays, and computational approaches. mpMRI has an important role in lesion detection and localization [6], while PSMA-PET provides complementary molecular imaging information in appropriate clinical settings [7]. Biomarker approaches such as the Prostate Health Index (PHI), 4Kscore, PCA3, SelectMDx, and ExoDx contribute additional information for risk stratification and biopsy decision-making [8,9,10,11]. Artificial intelligence-assisted approaches further illustrate the growing role of computational analysis in imaging and pathology [12]. The principal message is that no single test captures the biological and clinical heterogeneity of prostate cancer. Instead, precision diagnosis increasingly depends on combining molecular biomarkers with imaging, histopathology, and clinical risk information. Such integration may improve characterization of clinically significant disease while reducing unnecessary invasive procedures, although the performance and clinical utility of individual biomarkers remain dependent on the population, assay, and clinical context.

This diagnostic framework also provides context for the two epigenetic contributions in the Special Issue. Rather than treating methylation as a single generic biomarker class, these studies examine two complementary questions: how epigenetic regulation contributes to prostate biology and how methylation heterogeneity affects the analytical performance of molecular assays.

Baryshev and Vjaters investigated allele-specific methylation of the PSA promoter and its relationship to PSA expression across different prostate cell phenotypes [13]. The study identified distinct methylation patterns in normal prostate epithelial cells, benign prostatic hyperplasia cells, indolent prostate cancer cells, and aggressive prostate cancer cells. These findings support the concept that variation in PSA expression detectable in the circulation is influenced not only by genetic regulation of the PSA gene but also by epigenetic regulation through CG/CCWGG methylation. The importance of this work is therefore primarily mechanistic. By linking allele-specific promoter methylation with differences in PSA expression across prostate-cell phenotypes, the study provides a biological basis for further investigation of methylation as a molecular marker. The findings do not by themselves establish clinical diagnostic performance; rather, they provide a rationale for future studies evaluating whether PSA promoter methylation can contribute to molecular discrimination among benign, indolent, and aggressive prostate disease [14].

Visser et al. address a different but closely related problem: the analytical consequences of heterogeneous DNA methylation for quantitative methylation-specific PCR (qMSP) [15]. Using synthetic DNA constructs with defined methylation patterns together with methylation analyses of prostate tissue and urine-derived material, the investigators showed that partially methylated DNA fragments can alter amplification efficiency, fluorescence intensity, and cycle-threshold values. Importantly, the position of unmethylated CpG sites within primer and probe binding regions can substantially influence assay performance. This study highlights a critical principle for liquid-biopsy biomarker development: biological heterogeneity can become an analytical variable. A methylation assay may therefore produce an apparently negative or attenuated signal even when biologically relevant methylation is present, depending on the underlying methylation architecture and the design of the primer and probe system. These observations are particularly relevant to urine-derived DNA, where the molecular material available for analysis may be heterogeneous and fragmented.

**Figure 1:**
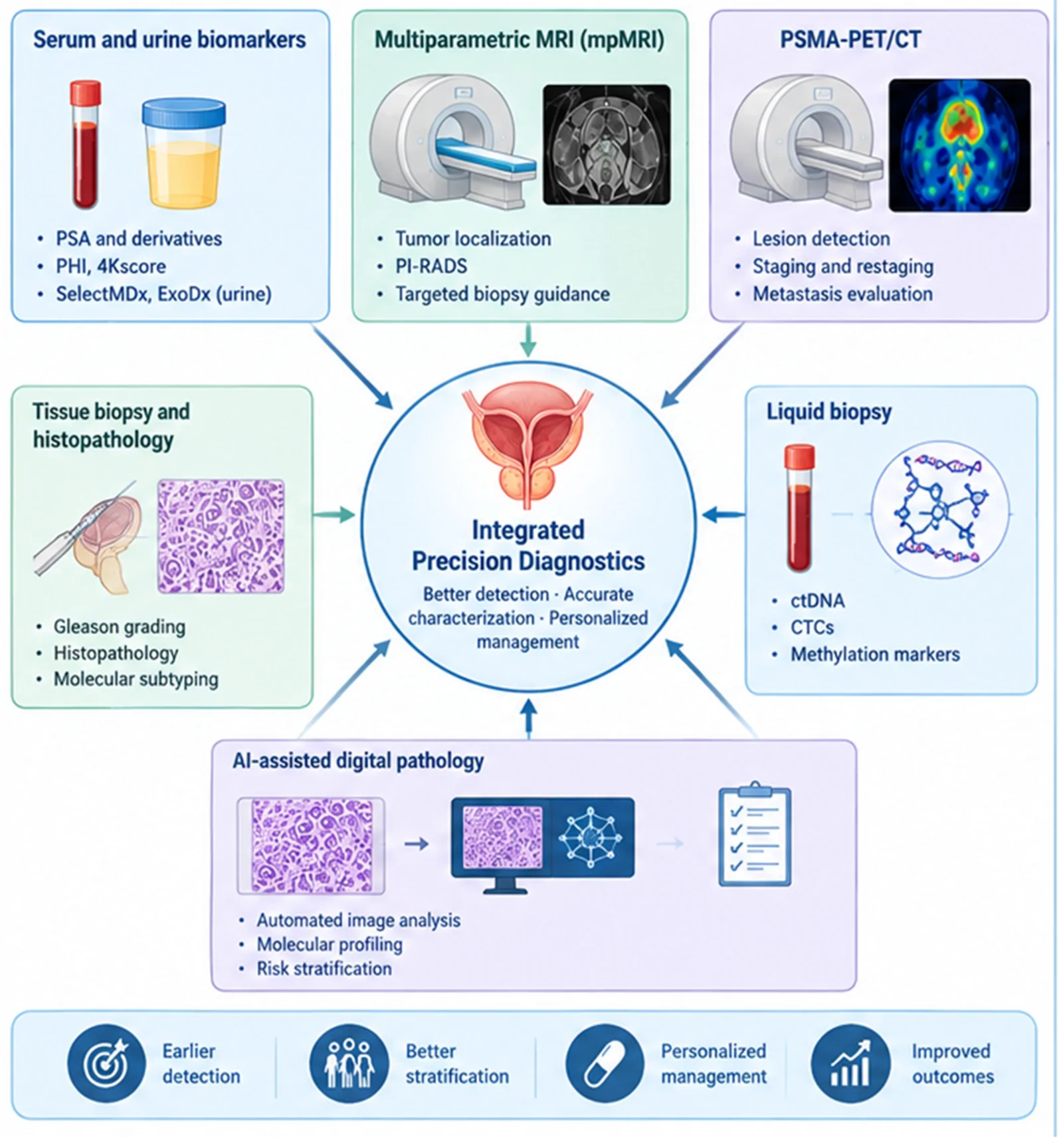
Contemporary precision diagnostic ecosystem in prostate cancer. Integrated molecular diagnostics combine serum and urine biomarkers, multiparametric magnetic resonance imaging (mpMRI), prostate-specific membrane antigen positron emission tomography/computed tomography (PSMA-PET/CT), tissue biopsy and histopathology, liquid biopsy, and artificial intelligence (AI)-assisted digital pathology to improve the detection, characterization, and risk stratification of clinically significant prostate cancer. The integration of genomic, epigenetic, imaging, and computational information may support more precise diagnosis, reduce unnecessary biopsies, and facilitate personalized management strategies across the disease continuum.

Together, the two methylation studies demonstrate complementary dimensions of epigenetic precision medicine. The PSA promoter study addresses the biological regulation of gene expression, whereas the GSTP1 study addresses the measurement of heterogeneous methylation in clinical specimens. Viewed together, they suggest that future methylation-based diagnostics will require both biologically informative marker selection and analytically robust assay design. Figure 1 summarizes data from reviews and articles addressing epigenetics within the context of the modern ecosystem of precision diagnostics for prostate cancer.

Genomic heterogeneity represents another central component of prostate cancer precision medicine. Pedroza-Torres et al. investigated androgen receptor-related genes using deep sequencing in a Mexican Hispanic prostate cancer cohort, addressing an important gap in genomic studies that have historically included populations of European ancestry disproportionately [16]. The study identified thousands of genomic variants, including alterations involving AR, SPOP, TP53, FOXA1, and MTOR, and reported 19 previously undescribed mutations in the studied cohort. Structural analyses suggested that several novel AR variants may influence receptor conformation within the amino-terminal domain, providing hypotheses for their possible functional consequences. Alterations in FOXA1 were also associated with poorer survival in the analyzed cohort. These observations should be interpreted as evidence for molecular diversity and candidate mechanisms rather than as definitive clinical biomarkers, because functional validation and larger population studies are required to establish the diagnostic, prognostic, or therapeutic significance of individual variants.

The broader contribution of this study is therefore both biological and methodological. It demonstrates that population composition matters when defining the genomic landscape of prostate cancer and reinforces the need to include diverse ancestral populations in precision-oncology research. Expanding genomic datasets can improve the representativeness of biomarker discovery and may help identify molecular features that would otherwise remain undercharacterized.

**Figure 2:**
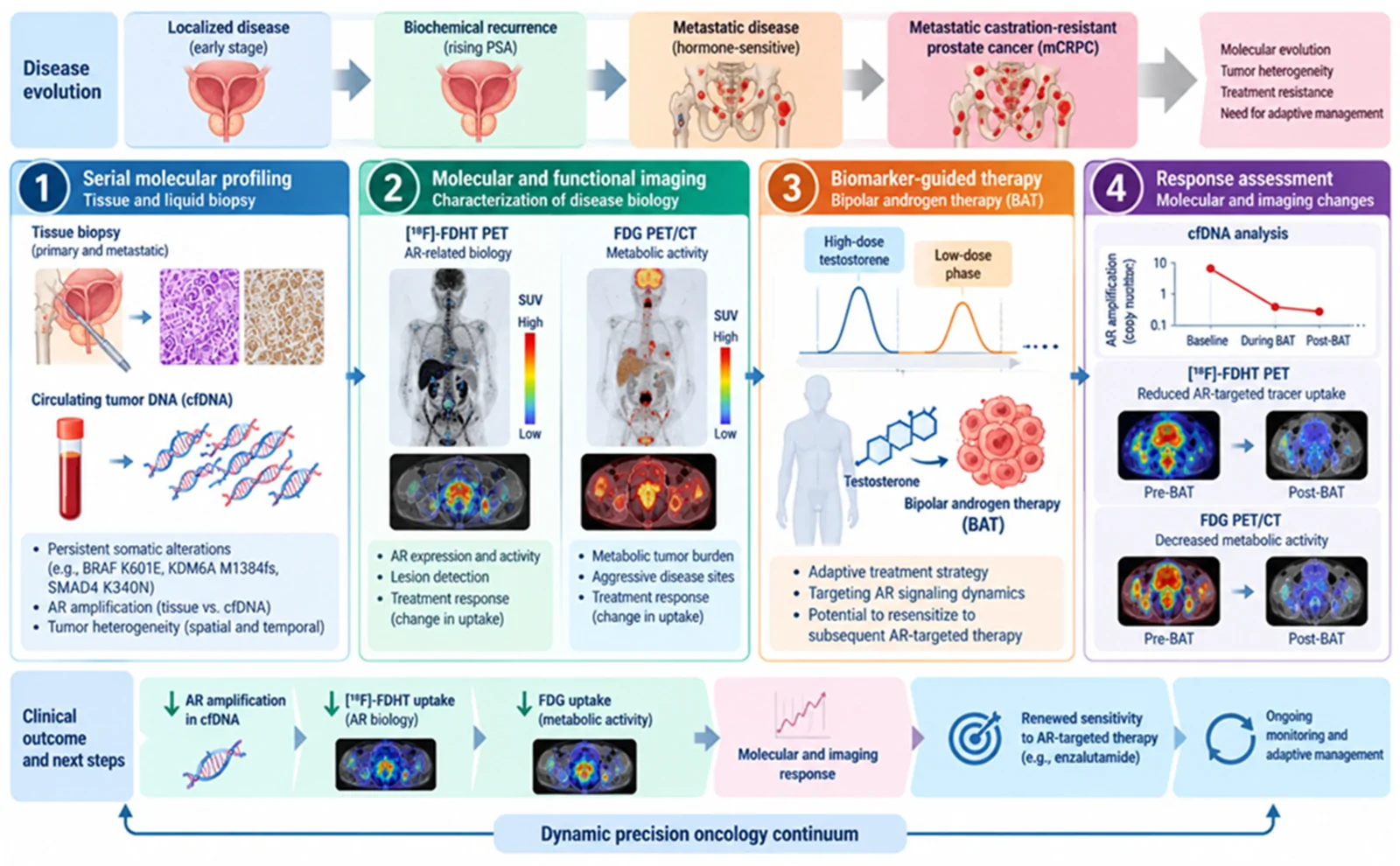
Longitudinal biomarker-guided precision management in metastatic prostate cancer. Precision oncology increasingly relies on repeated integration of molecular, liquid-biopsy, and imaging information as prostate cancer evolves. Serial tissue sampling and circulating cell-free DNA (cfDNA) analysis can identify persistent molecular alterations while revealing spatial and temporal tumor heterogeneity, including differences in androgen receptor (AR) amplification between disease sites. In the longitudinal case presented in this Special Issue, molecular profiling was integrated with [18F]-FDHT and FDG PET imaging to characterize AR-related and metabolic disease biology and to inform bipolar androgen therapy (BAT). Subsequent molecular and imaging assessments were used to evaluate treatment response and guide further clinical decision-making. The framework illustrates a dynamic precision-oncology process in which disease characterization, treatment selection, and response assessment are repeatedly reassessed as disease biology evolves.

The clinical case reported by Erazo et al. provides a particularly clear illustration of precision oncology as a longitudinal process rather than a single diagnostic event [17]. The report follows more than two decades of disease evolution and integrates serial tissue sampling, circulating cell-free DNA (cfDNA) analysis, and molecular imaging to characterize changing disease biology (Figure 2). Three somatic mutations—BRAF K601E, KDM6A M1384fs, and SMAD4 K340N—were consistently detected across the primary tumor, metastatic lesions, and cfDNA, demonstrating molecular continuity across disease sites. At the same time, differences in androgen receptor (AR) amplification between sampled sites highlighted intrapatient tumor heterogeneity. These findings illustrate both the value and limitations of liquid biopsy: cfDNA can provide repeated, minimally invasive molecular information, but its interpretation must consider spatial heterogeneity and the biological contribution of different tumor sites. The longitudinal assessment was further integrated with [18F]-FDHT and FDG PET imaging. [18F]-FDHT provided information related to AR expression, while FDG PET assessed metabolic activity. These complementary measurements contributed to the clinical interpretation of disease biology and supported the use of bipolar androgen therapy (BAT). Following BAT, the case demonstrated reductions in AR-related molecular and imaging signals together with a marked reduction in metabolic disease activity, followed by renewed sensitivity to androgen receptor inhibition.

This report should not be interpreted as evidence that BAT or any single biomarker strategy is universally effective. Rather, its value for this Special Issue lies in demonstrating how repeated molecular and imaging measurements can be integrated into individualized clinical decision-making. The case therefore provides a practical example of adaptive precision oncology in which disease characterization, treatment selection, and response assessment are linked over time.

Another contribution addresses precision oncology from the perspective of medicinal chemistry. Zyk et al. review approaches to the synthesis of DCL-based PSMA ligands, providing a chemical foundation for PSMA-directed molecular imaging and targeted radionuclide therapy [18]. The review examines synthetic strategies for protected DCL urea scaffolds, linker architecture, aromatic substitutions, and conjugation with diagnostic or therapeutic payloads. The authors also discuss synthetic routes relevant to clinically important PSMA-targeted compounds, including PSMA-11, PSMA-617, and Pylarify™, and propose an algorithm for selecting synthetic strategies according to ligand structure. This chemistry-focused perspective complements the diagnostic and clinical contributions of the Special Issue by demonstrating that theranostic medicine depends not only on target biology and imaging but also on the molecular design, synthesis, and physicochemical properties of targeting ligands.

PSMA provides a clear example of the theranostic principle: the same molecular target can support imaging-based disease localization and, when appropriately coupled to a therapeutic radionuclide, targeted treatment. Future development will require continued optimization of ligand affinity, pharmacokinetics, linker properties, payload attachment, and the relationship between target expression and therapeutic exposure.

## 2. From Biomarkers to Dynamic Precision Oncology

When considered together, the six contributions form a coherent precision-oncology continuum rather than a collection of unrelated topics. The diagnostic review begins with the question of how prostate cancer can be detected and characterized more accurately. The two methylation studies then demonstrate that molecular information has both biological and analytical dimensions: methylation can regulate gene expression, but heterogeneous methylation can also affect the reliability of the assay used to measure it. The genomic study extends this framework by showing that molecular diversity is also population-dependent and that underrepresented populations may harbor variants that remain insufficiently characterized in existing datasets. The longitudinal case then moves from characterization to management, illustrating how serial tissue, cfDNA, and molecular imaging can be combined as the disease evolves. Finally, the PSMA ligand review connects molecular target selection to the chemical development of imaging and therapeutic agents.

Across these contributions, a common principle emerges: precision oncology is increasingly dynamic. A molecular profile obtained at one point in the disease course may not fully represent subsequent tumor states, and the clinical meaning of a biomarker depends on the biological context, assay characteristics, disease site, and treatment history. Accordingly, future precision strategies are likely to rely increasingly on multimodal integration rather than isolated biomarkers.

Several priorities follow from this framework. First, molecular assays require rigorous analytical validation, particularly when heterogeneous DNA or methylation patterns can influence measurement. Second, genomic studies should include diverse populations to improve the generalizability and equity of precision medicine. Third, longitudinal sampling and imaging should be evaluated as complementary sources of information for monitoring tumor evolution and treatment response. Finally, theranostic development will benefit from coordinated advances in molecular targeting, medicinal chemistry, imaging, and therapeutic delivery. The integration of these elements may ultimately support a model in which prostate cancer is not characterized once, but repeatedly and contextually throughout its clinical course. Such a framework places the patient, rather than any individual biomarker or technology, at the center of precision decision-making. Precision oncology in prostate cancer is therefore moving from single-marker assessment toward integrated and longitudinal interpretation of molecular, epigenetic, genomic, imaging, and therapeutic information.

## 3. Conclusions

The six articles in this Special Issue illustrate complementary advances at the interface of molecular biology, epigenetics, genomics, imaging, medicinal chemistry, and clinical oncology. Collectively, they support a transition from conventional PSA-centered assessment toward integrated precision strategies that combine molecular diagnostics, epigenetic and genomic characterization, liquid biopsy, longitudinal monitoring, adaptive treatment, and PSMA-targeted theranostics.

Importantly, that clinical translation depends on analytical validity, appropriate population representation, longitudinal interpretation, integration of multimodal data, and careful validation of clinical utility. The Special Issue therefore provides a useful snapshot of how molecular information can increasingly be incorporated into individualized prostate cancer management while highlighting the work that remains before emerging biomarkers and strategies can be routinely implemented. Continued collaboration among molecular scientists, chemists, radiologists, pathologists, bioinformaticians, and clinicians will be essential for translating these advances into clinically meaningful and equitable precision medicine.

## Data Availability

No new data were created or analyzed in this editorial.
